# Impact of Dental Fluorosis, Socioeconomic Status and Self-Perception in Adolescents Exposed to a High Level of Fluoride in Water

**DOI:** 10.3390/ijerph14010073

**Published:** 2017-01-12

**Authors:** Nelly Molina-Frechero, Martina Nevarez-Rascón, Alfredo Nevarez-Rascón, Rogelio González-González, María Esther Irigoyen-Camacho, Leonor Sánchez-Pérez, Sandra López-Verdin, Ronell Bologna-Molina

**Affiliations:** 1 Departamento de Atención a la Salud, Universidad Autónoma Metropolitana (UAM) Unidad Xochimilco, Calzada del Hueso 1100, 04900 Ciudad de México, Mexico; nmolina@correo.xoc.uam.mx (N.M.-F.); meirigo@correo.xoc.uam.mx (M.E.I.-C.); tlsperez@correo.xoc.uam.mx (L.S.-P.); 2 Facultad de Odontología, Universidad Autónoma de Chihuahua, (UACH), Campus I Av. Universidad s/n, 31000 Chihuahua, Mexico; martina.nevarez@gmail.com (M.N.-R.); alfredonevarez@hotmail.com (A.N.-R.); 3 Facultad de Odontología, Universidad Juárez del Estado de Durango (UJED), Predio Canoas s/n, 34000 Durango, Mexico; rogegg@hotmail.com; 4 Centro Universitario de Ciencias de la Salud, Universidad de Guadalajara (UdeG), Sierra mojada 950, 44340 Guadalajara Jalisco, Mexico; patologiabucal@live.com.mx; 5 Facultad de Ciencias de la Salud, Universidad Anáhuac Norte, Av. Universidad Anáhuac 46, 52786 Estado de Mexico, Mexico; ronellbologna@hotmail.com

**Keywords:** self-perception, dental fluorosis, Thylstrup and Fejerkov index, adolescents

## Abstract

*Objective*: To identify adolescents’ self-perception of dental fluorosis from two areas with different socioeconomic levels. *Methods*: A cross-sectional, descriptive study was conducted with 15-year-old youths by applying a questionnaire designed and validated to assess self-perceptions of dental fluorosis in two areas with different socioeconomic statuses (SESs). Fluorosis was clinically evaluated by applying the Thylstrup and Fejerkov (TF) index on the upper front teeth. *Results*: A total of 308 adolescents were included in the study. The medium-SES population, which was exposed to 2.5 ppm of fluoride in water, and the low-SES population, which was exposed to 5.1 ppm, presented the following levels of dental fluorosis: TF 2–3 (50%), TF 4–5 (45.6%) and TF 6–7 (4.4%) for medium SES and TF 2–3 (12.3%), TF 4–5 (67.1%) and TF 67 (20.6%) for low SES. A significant association was found between self-perception and dental fluorosis in those with medium and low SESs (*p* < 0.05). The multiple regression model found differences between TF levels and self-perception, with a 6–7 TF level for concerns about color (OR = 1.6), smile (OR = 1.2) and appearance (OR = 3.36). *Conclusions*: Self-perceptions of dental fluorosis affect adolescents such that adolescents with a medium SES have more negative perceptions than those with a low SES. Such perceptions increase as the TF index increases.

## 1. Introduction

Dental fluorosis is a hypomineralization of the enamel that is characterized by greater porosity of the surface of healthy enamel due to excess fluoride consumption during the odontogenesis period. Defects in the structure of dental enamel are characterized by hypocalcified areas with spots of hypoplasia, which cause the loss of the enamel structure upon spreading and could lead to the loss of the tooth shape [1]. The enamel changes in color from fine white lines to stained enamel. Depending on the degree of severity, abnormalities in the coloration of the teeth and dark stains with yellow to chocolate-brown coloring affect the appearance of young people because the smile is the first impression of one’s teeth [2].

As a very important part of dental aesthetics and facial appearance, the smile can greatly affect how people perceive their overall attractiveness. The smile is the second facial feature that people tend to note for attractiveness in others; only the eyes are more important. Therefore, dental aesthetics can greatly impact someone’s attractiveness [2]. In adolescents, smiling has a significant impact on self-esteem. As adolescents interact with their environment, smiling indicates self-confidence and well-being [2,3].

The adolescent stage is when youths are concerned about their appearance, and stained teeth, especially those expressed in a smile (the upper front teeth), are the first to be noticed.

Concerns about color cause problems when smiling, which create two factors that can affect one’s appearance: showing insecurity when teeth are visible through a smile and feeling negatively affected by this appearance. In other words, what could be called self-perception is a psychological characteristic of individuals and is fundamental to what an individual feels about his/her own appearance, including experiencing psychosocial suffering that affects his/her happiness [4,5].

Studies conducted in developed countries have attempted to identify the possible psychological impact of this condition in adolescents by exploring developmental defects of the enamel and its influence on everyday life. The majority of these studies have been conducted in populations with mild levels of fluorosis [6,7].

The state of Durango is an endemic area for dental fluorosis, as it affects nearly the entire population. Reports by the Secretary of Health indicate the high prevalence of fluorosis and the severity of this issue [8]. This disorder is a serious public health problem, and this severity indicates the seriousness of dental disorders. Durango City is home to most of the state’s population. The concentration of fluoride in the water ranges from 2.2 to 7.2 ppm [9], leading to a condition that seriously compromises the appearances and health of individuals affected by the most severe levels.

Given the lack of knowledge about the influence of this disorder at the dental level in youth and given that this life stage places great importance on appearance, understanding the degree of dental fluorosis and associating it with self-perception in adolescents in areas with different socioeconomic statuses (SESs) will help establish behavioral parameters that will be useful for establishing future medical, dental and psychological therapies associated with fluorosis. Thus, the purpose of this study was to identify the severity of fluorosis in two groups of adolescent youths from different SESs and to relate this severity to self-perception.

## 2. Materials and Methods

Study design: This cross-sectional descriptive and analytical study was conducted with a sample of adolescents from an endemic area for dental fluorosis with different socioeconomic levels according to the CONAPO (Consejo Nacional de Población) [10] criteria. The data were collected from the city of Durango from January 2014 to June 2015. The study was approved by the Universidad Juárez del Estado de Durango’s ethics committee (Code: UJED FOD 12).

Study population: The study involved 15-year-old adolescents of both genders from secondary schools representative of the study areas. The majority of the adolescents were from two SES groups (medium and low SES).

Differentiation of socioeconomic status: medium SES corresponds to the medium-high level; thus, there is no upper-SES class, in contrast to other states. The predominant population of average purchasing power comprises families with well-paying jobs, middle-income levels and medium to high levels of education, and this group is characterized by professionals, entrepreneurs and business people. The low-SES level comprises low-income families without permanent jobs who are dedicated to construction and informal businesses and who have little education and larger families [10].

The schools were selected using a model of population proportion with a confidence interval of 95% and a margin of error of 7% based on a standard deviation that was obtained from a previous study. Adolescent from six schools were selected: three of medium SES and three of low SES.

**Figure 1:**
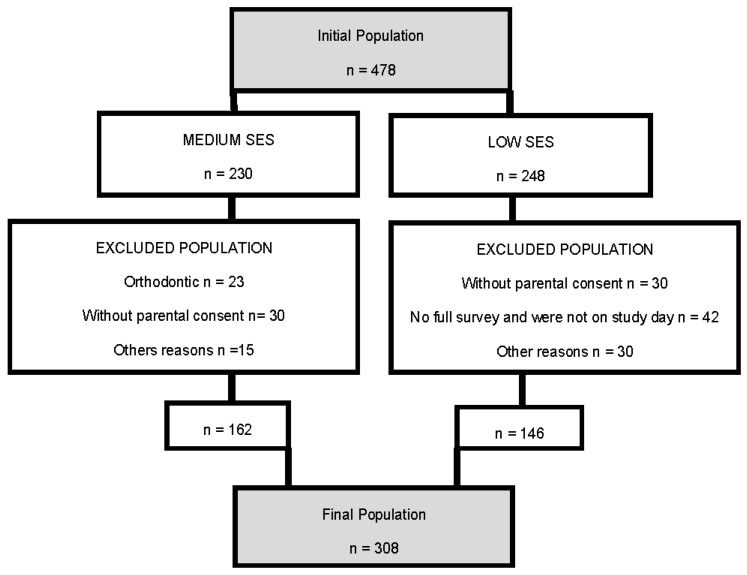
Information on the sample size in a flow diagram

Sample size: The sample from the schools selected for the study included 478 adolescents, corresponding to 20% of the population of that school age. A total of 230 adolescents were from the (area considered medium SES, and 248 were from the area considered low SES. Finally, 308 youth who submitted an authorization form to participate in the study and met all of the inclusion criteria and participation requirements were included. For various reasons, 29.6% of the adolescents were excluded from the medium-SES group, and 41.2% were excluded from the low-SES group. The sample comprised 162 adolescents from the medium-SES group and 146 from the low-SES group (Figure 1).

Criteria: The study included 15-year-old adolescents enrolled in secondary schools in the study area who submitted signed authorization forms from their parents or guardians and who were in attendance on the days when the study was performed. Adolescents who were excluded were students who did not meet the inclusion criteria and adolescents who had dental caries, malocclusion in the front upper teeth or other issues that could affect the appearance of their smiles. Others were excluded because they were not cooperative during the review or because they submitted surveys that were unreliable, incomplete, confusing or difficult to read.

The independent variables were gender, dental fluorosis and SES. The dependent variable was self-perception of appearance, which was assessed through the variables of concern about the color of one’s teeth, problems when smiling and issues with the appearance of the teeth.

A questionnaire was used to explore the sociodemographic variables of the study participants and to assess their self-perception. Simple questions for understanding young people were developed and designed, with three of the questions based on the studies by Van Palenstein Helderman W.H. and Mkasabuni E. [11] and Riordan [12] concerning the impact of dental fluorosis on dental aesthetics: Are you concerned about the color of your teeth? Do you have trouble smiling because of your teeth? Are you concerned about the appearance of your teeth? These questions were asked with five possible Likert-type responses that were later categorized as affirmative or negative (Yes or No). An assessment was conducted to determine the questionnaire’s consistency and the criterion validity of the questions in order to identify whether adolescents’ self-perceptions were determined by their teeth when affected by dental fluorosis. This assessment occurred through a pilot test with 30 adolescents from secondary schools neighboring the study areas. Values > 0.7 were obtained using Cronbach’s alpha coefficient. The reliability of the questionnaire was calculated using an intraclass correlation that yielded values greater than 70% [13,14].

Analysis of the water: Prior to the study, an analysis of the fluoride concentration in the water of the two study areas was conducted using the Orion EA 920 model potentiometer with an ion-specific electrode (Model 9409 BN, Orion, Cambridge, MA, USA) [15].

Clinical evaluation of dental fluorosis: An oral evaluation of the labial surfaces of the front upper teeth from canine to canine was conducted by two evaluators calibrated according to the World Health Organization [16] criteria.

Standard infection control measures were followed using epidemiological exams. The teeth were dried for the exam, which was completed in daylight but not in direct sunlight by applying the Thylstrup and Fejerskov index [17,18] and recording the range found.

Two examiners participated in a pre-study calibration exercise with the Thylstrup and Fejerskov index. Intra-examiner reliability was assessed, and kappa coefficients of >0.96 and >0.88 were found for inter-examiner reliability and for severity, indicating that all participants had some level of dental fluorosis.

Statistical analysis. The collected information was removed and coded in a database on the same day that the survey was completed. The IBM SPSS 21 program (IBM, Armonk, NY, USA) was used for data collection. The analysis was conducted with descriptive statistics and bivariate and multivariate analyses. The univariate analysis used means, standard deviations and 95% confidence intervals. The bivariate analysis used contingency tables, chi-square tests and odds ratios (ORs) with their respective 95% confidence intervals. A logistic regression model was constructed.

## 3. Results

The water analysis was conducted in 16 wells corresponding to each study area, yielding a mean of 2.51 ± 0.35 ppm in the medium-SES area and 5.14 ± 1.03 ppm in the low-SES areas.

The study included 308 adolescents, 53.46% of whom were female. The entire population showed signs of dental fluorosis distributed on the Thylstrup and Fejerkov (TF) index as TF 2–3 (32.1%), TF 4–5 (55.8%) and TF 6–7 (12%), with a mean of 4.13 (CI 95%: 4.0–4.26).

**Figure 2:**
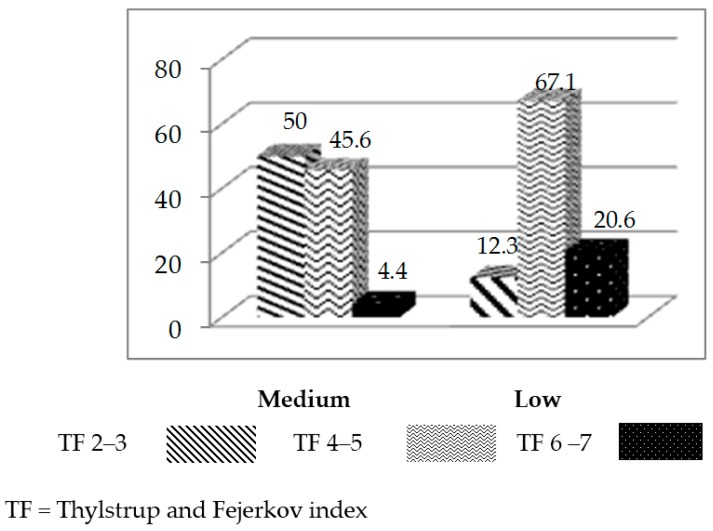
Severity of dental fluorosis in adolescents with medium and low SES in percentages.

The medium-SES area included 162 adolescents, 50% of whom had TF 2–3, 45.6% of whom had TF 4–5, and 4.4% of whom had TF 6–7. In the low-SES area, the sample included 146 adolescents, 12.3% of whom had TF 2–3, 67.1% of whom had TF 4–5, and 20.6% of whom had TF 6–7. These results show differences in the levels of severity (χ^2^ = 57.060, *p* = 0.000) (Figure 2).

### 3.1. Concerns about Color

In the TF 2–3 group, 55 (68%) adolescents from the medium-SES group expressed concerns about tooth color, whereas only seven (39%) adolescents in the low-SES group expressed this concern (*p* = 0.022) (Table 1).

In the TF 4–5 group, 56 (76%) medium-SES adolescents and 59 (60.2%) low-SES adolescents were concerned about color (*p* = 0.024) (Table 2).

In the TF 6–7 group with the greatest severity of dental fluorosis, only seven youths were from the medium-SES group, and all of them expressed concerns about color. In the low-SES group, five out of 30 adolescents (16.6%) were not concerned about tooth color (Table 3).

### 3.2. Concerns about Smile

In the TF 2–3 group, 26.9% of the adolescents from the medium-SES group expressed concerns about smiling, whereas 27.8% of the adolescents in the low-SES group expressed this concern, indicating no significant difference (*p* = 0.541) (Table 1).

In the TF 4–5 group, 75.7% of the medium-SES students were concerned about smiling, whereas 57.1% of the low-SES students expressed this concern (*p* = 0.009) (Table 2).

In the TF 6–7 group with the greatest severity of dental fluorosis, only seven youths were from the medium-SES group, and they all expressed concerns about smiling. In the low-SES group composed of 30 adolescents, five (16.6%) did not express concerns about smiling (Table 3).

In the two socioeconomic levels, the adolescents showed significant differences in concerns about smiling. In the low-SES group, a greater number of adolescents expressed that they could smile despite having high levels of fluorosis relative to the number of adolescents in the medium-SES group, where the percentage of concern was greater.

### 3.3. Concerns about Appearance

In the TF 2–3 group, 74.4% of the adolescents from the medium-SES group expressed concerns about the appearance of their teeth, whereas 38.8% of the low-SES group expressed this concern, indicating a significant difference (*p* = 0.013) (Table 1).

In the TF 4–5 group, 82.4% of the medium-SES adolescents were concerned about their appearance, whereas 59.6% of the low-SES adolescents had this concern (*p* = 0.000) (Table 2).

In the TF 6–7 group with the greatest severity of dental fluorosis, all of the adolescents in the medium-SES group expressed concerns about their appearance, whereas only one student in the low-SES group expressed a lack of concern about his/her appearance (Table 3).

There were no gender-related differences in concerns about color, smile and appearance (*p* > 0.05); concern was expressed in both gender groups.

Table 4 shows a logistic regression analysis using Wald statistics to interpret the B coefficient in terms of the OR and 95% confidence intervals for responses about the self-perception of dental fluorosis at different TF levels. In the ranges of greatest severity (TF 6–7), we found more than three times the concern for appearance, whereas the concern was 2.8-fold greater in the TF 4–5 group.

## 4. Discussion

Half of the study population had dental fluorosis at the TF 4–5 level. The entire surface exhibits marked opacity or appears chalky white. Parts of the surface exposed to attrition appear less affected, and at level 5, the entire surface displays marked opacity with a focal loss of the outermost enamel (pits) <2 mm in diameter [17]; this involved staining of the entire enamel structure by extrinsic pigmentation that appeared yellow, dark brown and chocolate in color. Furthermore, this enamel issue can present small areas of hypoplasia, which can appear as fractures of the incisal edge of the front teeth.

The youths from the medium-SES group had less severe dental fluorosis, constituting the lowest TF values and including fewer individuals with high severity, whereas the low-SES group tended to have greater concerns about their teeth. Such differences based on SES were significant (*p* < 0.05).

All of the adolescents in the study had resided in the area endemic for fluorosis since birth. As such, they were accustomed to seeing teeth with clinical characteristics of fluorotic enamel. The perception of aesthetics in adolescents from the medium-SES group was greater in those with the lowest levels of TF severity, manifesting as concerns about color (OR = 1.262) and appearance (OR = 1.299). This finding could be compared to the results of other studies conducted in Western countries, where concerns about aesthetics arise even from low levels of dental fluorosis, such as white chalky coloring, and where adolescents consider their teeth to be unattractive and express insecurities about smiling [6,19].

A study conducted by Sigurjons et al. [20] in the United Kingdom found that parents of children with teeth in the TF 3 range expressed displeasure about the color and appearance of their children’s teeth. Another study reported that children felt ashamed and parents expressed displeasure about the color of their children’s teeth, concluding that this level of fluorosis was unacceptable [11]. Spots whose color differs from the normal shade of enamel capture people’s attention, resulting in poor aesthetic acceptability. In Mexico City, a study was conducted on children who went to a university dental clinic and expressed concern about the color of their teeth [21,22].

The group of adolescents from the low-SES group with TF 2–3 expressed less concern and greater acceptability for teeth with whitish spots because the color did not pose aesthetic problems for them. Differences in SES arose because these groups had different interpretations of dental appearance (*p* < 0.05). This finding was likely due to the environment because low-SES youth were born and raised in conditions where all of their acquaintances had stained teeth, which resulted in more favorable self-perceptions.

At the TF 4–5 level in the two groups, we found that concerns regarding dental appearance increased as the scores or measures of severity of the TF index increased. Medium-SES adolescents were more likely to have a negative self-perception related to their concerns about color (OR = 1.542), smile (OR = 1.667) and appearance (OR = 2.206). These results are similar to the findings of the 2005 study by Edwards et al. [23], who used a web simulator to measure perceptions about dental fluorosis in adolescents and found that acceptability decreased as the level of dental fluorosis became more severe.

In recent decades, dentistry has recognized the aesthetic, emotional and psychological problems related to one’s appearance as an important aspect of health. An altered dental appearance can cause problems for young people starting in childhood as a result of the ridicule they experience from classmates, and these problems manifest as low self-esteem [22,23,24].

Additionally, it is well known that physical appearance has a great psychosocial impact on a large portion of the population. Moreover, it is recognized that the mouth and teeth play a crucial role in verbal and non-verbal communication and constitute a significant element of social interactions. The color of teeth is also perceived as critical in one’s satisfaction with the appearance of the smile [9,25,26].

Studies conducted in Brazil by Elwood [27] and Silva da Castilho et al. [28] indicate that young people who feel concerned about the color of their teeth and embarrassed about smiling tend to exclude themselves. Brazilian youth expressed greater acceptance than youth in the United Kingdom with similar levels of dental fluorosis.

Although the importance of dental aesthetics and the psychosocial repercussions have been emphasized, few studies have been conducted on the aesthetic impact of fluorosis in endemic areas and areas with high severity levels. This scarcity is probably due to the existence of other social and cultural factors that are predominant in those regions. In India, various studies [29,30,31] have been conducted on adolescents exposed to concentrations above 4 ppm and on those who showed high levels of severity; these studies have found that young people in India perceive fluorosis as an aesthetic problem that causes problems with smiling. Despite this concern, the study by Naidu et al. [30] found that a significant percentage of youth showed no awareness of this problem, perhaps because they belonged to a low socioeconomic class.

In adolescents who had a higher severity level (TF 6–7), all students from the medium-SES group expressed concerns about color, smile and appearance, whereas some young people of low SES were not similarly concerned about color and appearance. We concluded that these results could be due to the socioeconomic levels of the adolescents.

The study had several limitations. The study had a cross-sectional design and could not establish relationships beyond associations, and the population of adolescents with a TF 6–7 severity level was insufficient. Another limitation was the lack of knowledge of the sociocultural factors of the study population.

## 5. Conclusions

The results indicate that anti-aesthetic colorations due to dental fluorosis affect adolescents and their psychosocial relationships. More severe dental fluorosis produces greater aesthetic concerns related to the color of the teeth, particularly when smiling, as this problem affects people’s appearance. SES plays an important role because adolescents with a medium SES have less exposure to and less severity of dental fluorosis and thus have greater dental concerns than do low-SES adolescents who face other concerns, such as economic concerns. Given their restricted options, preventive or restorative measures for aesthetic and functional changes are not considered. This issue causes different interpretations based on anxiety stemming from unattractive teeth and the impacts of a person’s social environment.

This condition constitutes a severe public health problem in which inequalities in living conditions lead to inequalities in one’s health. Institutions at the local, regional and national levels should implement preventive environmental and health measures based on the recognition that this issue is a multifactorial problem where the most socio-economically disadvantaged populations are at greatest risk and the finding that this issue affects both biological and social health.

## Figures and Tables

**Table 1:** Bivariate analysis between self-perception and different socioeconomic levels in the TF 2–3 population.

Concerns about Color	OR	CI 95%	*p* Value
Medium SES	1.262	1.005–1.585	0.022
Low SES			
**Concerns about Smile**			
Medium SES	0.983	0.792–1.219	0.541
Low SES			
**Concerns about Appearance**			
Medium SES	1.299	1.022–1.651	0.013
Low SES			

OR (odds ratio), CI (confidence interval).

**Table 2:** Bivariate analysis between self-perception and different socioeconomic levels in the TF 4–5 population.

Concerns about Color	OR	CI 95%	*p* Value
Medium SES	1.542	1.007–2.360	0.024
Low SES			
**Concerns about Smile**			
Medium SES	1.667	1.086–2.558	0.009
Low SES			
**Concerns about Appearance**			
Medium SES	2.206	1.330–3.658	0.000
Low SES			

OR (odds ratio), CI (confidence interval).

**Table 3:** Bivariate analysis between self-perception and different socioeconomic levels in the TF 6–7 population.

Concerns about Color	OR	CI 95%	*p* Value
Medium SES	0.781	0.650–0.938	0.327
Low SES			
**Concerns about Smile**			
Medium SES	0.767	0.650–0.934	0.198
Low SES			
**Concerns about Appearance**			
Medium SES	0.806	0.686–0.946	0.811
Low SES			

OR (odds ratio), CI (confidence interval).

**Table 4:** Logistic regression model (concerns about color, smile and appearance) in the degrees of fluorosis severity.

Self-Perception	Β	Wald	*p* Value
ITF 2–3			
Color	0.452	0.874	0.350
Smile	0.956	3.297	0.069
Appearance	0.454	0.874	0.350
ITF 4–5			
Color	1.856	10.245	0.001
Smile	1.982	11.719	0.001
Appearance	2.804	18.951	0.000
ITF 6–7			
Color	1.609	10.793	0.001
Smile	1.190	7.594	0.006
Appearance	3.367	10.961	0.001
